# A new era for African health systems: Market shaping and the African Continental Free Trade Area (AfCFTA)

**DOI:** 10.1002/puh2.172

**Published:** 2024-05-07

**Authors:** Jonta Kamara, Ukeme Essien, Alain Labrique

**Affiliations:** ^1^ School of Life Course and Population Sciences, Faculty of Life Sciences & Medicine King's College London, London, United Kingdom of Great Britain and Northern Ireland; ^2^ Multidisciplinary Adolescent and Youth Review Board Second Lancet Commission on Adolescent Health and Wellbeing Toronto Ontario Canada; ^3^ Department of Biostatistics Johns Hopkins Bloomberg School of Public Health Baltimore Maryland USA; ^4^ Department of International Health Johns Hopkins Bloomberg School of Public Health Baltimore USA

**Keywords:** drug industry, health care market, health policy, health system, intellectual property

## Abstract

The COVID‐19 pandemic has forced a reflection on the origins of supplies in African healthcare market and underscored the need for an increase in local manufacturing of medical supplies. Several African countries’ health markets have been heavily reliant on imports. First, this article demonstrates how the African healthcare market has had a high import dependency and the role that the African Continental Free Trade Area (AfCFTA) could play to reverse this. It is estimated that African countries import between 80% and 94% of medical supplies, 75% of testing kits, between 70% and 95% of pharmaceuticals, and 99% of vaccines. Second, during the COVID‐19 pandemic, countries imposed export restrictions which impacted the flow of medical supplies to African countries. This finding highlighted the limited production capabilities on the African continent and reiterated the need to strengthen continental value chains and local manufacturing capacity to establish the continent's New Public Health Order. Third, there was the emergence of local innovations seeking to minimize the impact of these supply chain disruptions. Using case studies on the local production of COVID‐19 testing kits and personal protective equipment, the article highlights progress made toward health market reform. It calls attention to the implementation of the AfCFTA to strengthen the supply, manufacturing, and trade of medical resources. Fourth, this article highlights countries that have African‐made pharmaceuticals and vaccinations and the importance of regional hubs to expand these products in African healthcare markets. It concludes by discussing investments made to expand local manufacturing of health products.

## Graphical abstract

A new era of African health systems is being established as countries have had a high medical import dependency. During the COVID‐19 pandemic, local Innovations were created, and through the implementation of the African Continental Free Trade Area (AfCFTA), investments in local manufacturing can help reverse this.

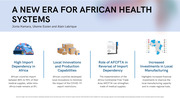

## INTRODUCTION

**FIGURE 1:**
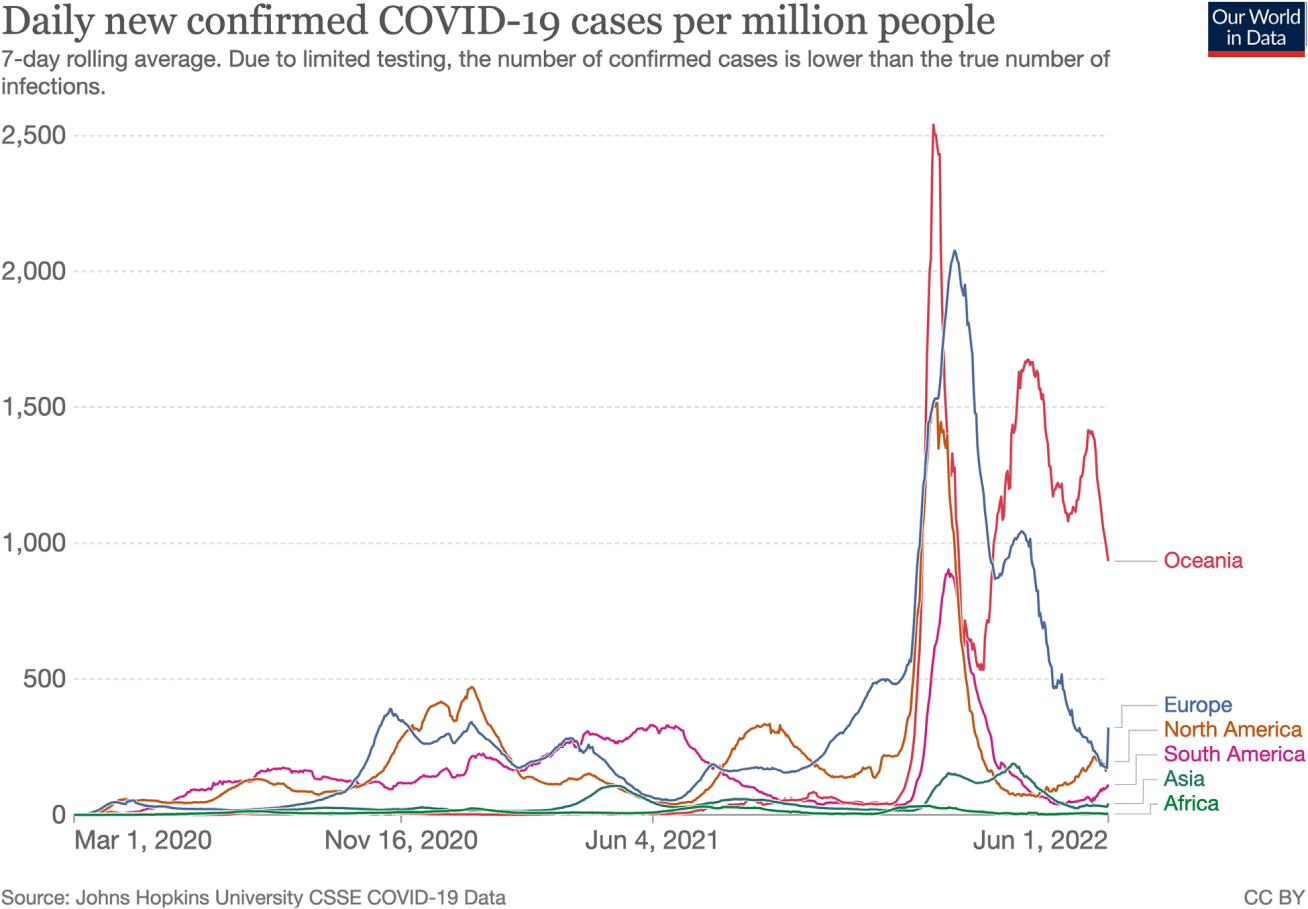
Epidemiological curve of COVID‐19 cases by continents. *Source*: Adapted from Ref. [9].

The first wave of the COVID‐19 pandemic unexpectedly spread slower on the African continent compared to the rest of the world as shown in Figure 1 [1]. This was contrary to fears that poor governance and weak healthcare infrastructure would make African countries the most vulnerable [2, 3, 4]. This concern arose from the experience around previous outbreaks, such as Ebola, which severely challenged the affected African countries. Experiences from previous outbreaks led to the official launch of the Africa Centre for Disease Control and Prevention (Africa CDC) in January 2017 [5, 6]. Its primary goal is to help African Union (AU) Member States improve their public health systems and thereby prevent the potential devastating impact of future outbreaks [7, 8]. Africa CDC applied lessons learned from Ebola and previous outbreaks to the COVID‐19 response.

**FIGURE 2:**
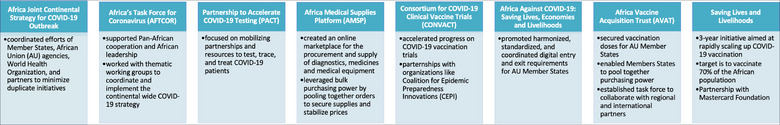
Initiatives launched by Africa Centres for Disease Prevention and Control (Africa CDC) during the COVID‐19 pandemic. *Source*: Adapted from Refs. [16, 17, 18].

The first reported case of COVID‐19 on the African continent was in Egypt on February 14, 2020 [10]. This was 2 weeks after the World Health Organization (WHO) declared the COVID‐19 Outbreak a Public Health Emergency of International Concern [11]. Due to minimal testing, there was a limited understanding of the full impact of the pandemic in African countries, particularly in the earlier months of its onset [12]. During the COVID‐19 pandemic, Africa CDC launched eight initiatives as part of its response, which are highlighted in Figure 2. It convened Ministers of Health and provided countries with strategic, technical, and logistical assistance to respond to the COVID‐19 pandemic [13]. It also had a central role in information sharing, procurement of medical supplies, and capacity building [14]. A key initiative was the Partnership to Accelerate COVID‐19 Testing, which trained 905 health workers and 499 were certified to use rapid antigen tests [15]. This made it possible to visit more than 2.5 million households for contact tracing, active case searching, and risk communication activities identifying more than 1.6 million contacts [15].

The AU estimates Africa's loss in economic growth due to the COVID‐19 pandemic at US$270 billion [19]. Namely, it has been challenging to make investments in health systems, due to rising debt levels in sub‐Saharan Africa (SSA), which increased by 58% during the COVID‐19 pandemic, according to the International Monetary Fund (IMF) [20]. This impact on economies especially debt poses challenges to investments in health systems.

## THE OVERRELIANCE ON MEDICAL IMPORTS

The COVID‐19 pandemic highlighted structural and systemic inequities creating barriers, for low‐ and middle‐income countries to access medical supplies [21]. On the African continent, key challenges are the limited diversification in medical supply chains and lack of production capability [21, 22]. It is estimated that Africa produces only 6%–20% of its medical products [23, 24]. The International Trade Centre (ITC) estimates that 80%–94% of the continent's medical needs are met through imports, whereas the IMF estimates stands at 85% [23, 24, 25]. This is in stark contrast to the European Union (EU) which only imports 6.6% of medical supplies and highlights vulnerabilities of the continent should trade restrictions be imposed [26].

**FIGURE 3:**
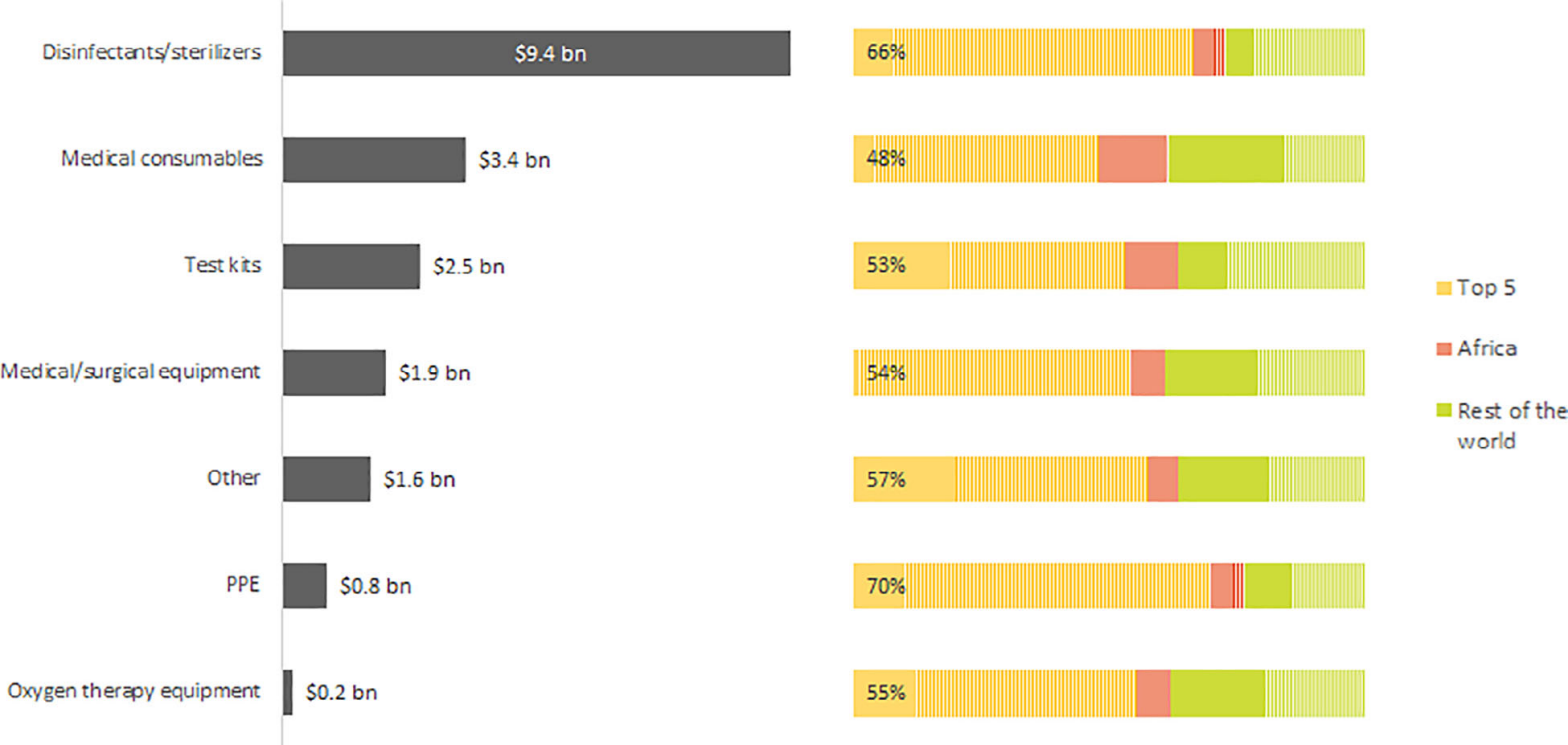
International Trade Center (ITC) medical supply imports in Africa as of May 2020. *Source*: Adapted from Ref. [23].

Figure 3 highlights the top five global exporters, including the EU, China, and India, which are responsible for providing 71% of personal protective equipment, 66% of disinfectants and products, and 48% of medical consumables imports in Africa [23, 25]. African countries have limited capacity to process raw materials locally, exporting up to 3.5% of raw materials needed globally to make masks, gloves, and disinfectants [23]. This limited ability to process these materials is one reason why countries rely heavily on imports.

**FIGURE 4:**
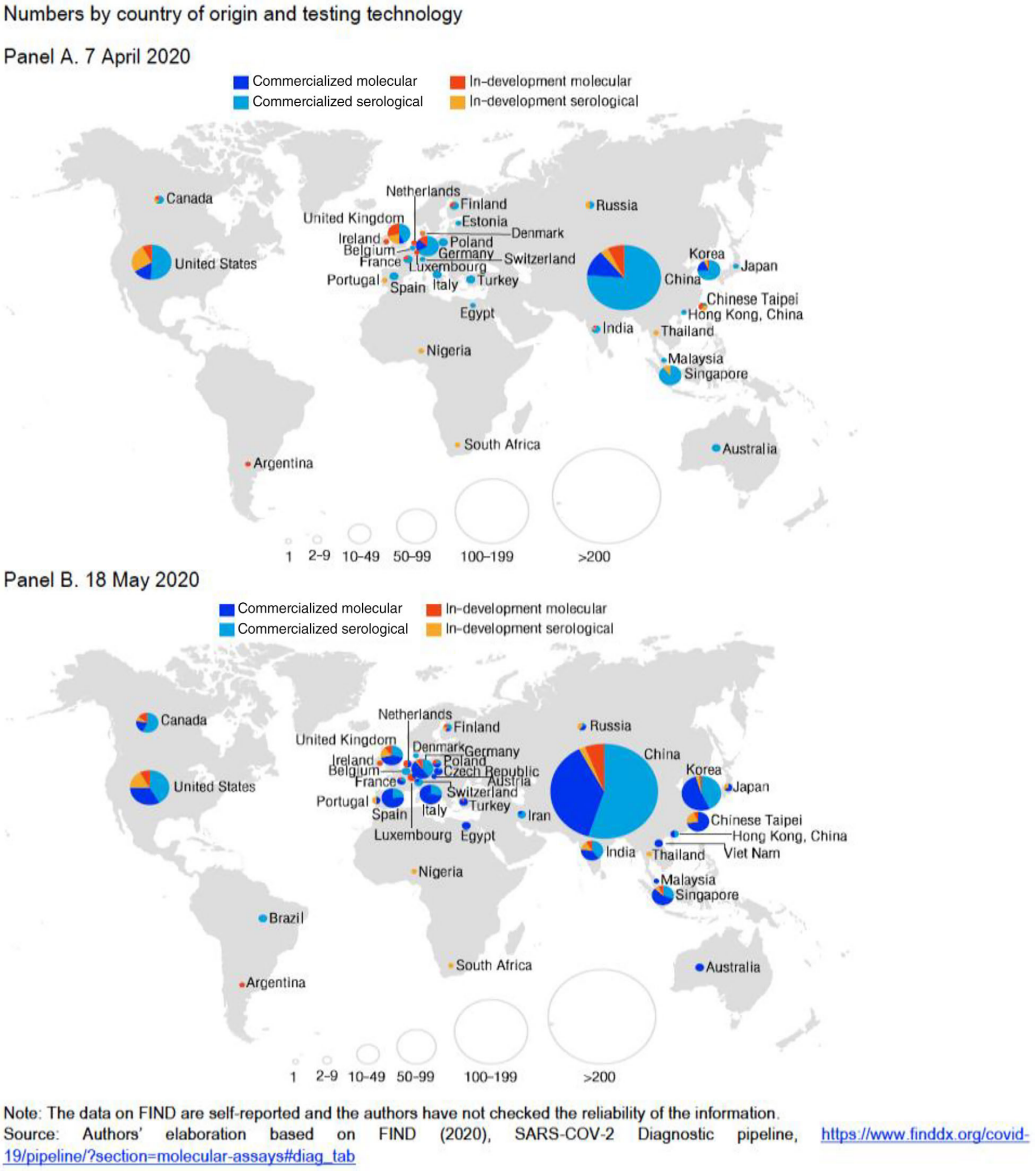
Number of commercialized or in‐development tests for COVID‐19 globally in April and May 2020. *Source*: Adapted from Ref. [28].

This issue of overreliance, coupled with limited domestic diagnostic test development infrastructure, led to insufficient capacity to scale‐up COVID‐19 testing. Restrictions by the EU which provides 65% of test kits to SSA, limited Africa's access to COVID‐19 test kits even when funds were readily available [25, 27]. At the beginning of the pandemic, Africa CDC held training sessions to ensure staff competency testing, but this did little to mitigate deeper manufacturing and supply chain issues [27]. Compared to the rest of the world, Africa's capacity to provide COVID‐19 testing kits was minimal as highlighted in Figure 4.

The disruption to supply chains also revealed the limited purchasing power of African countries. This is underscored by the Center for Global Development which showed decreased pharmaceutical exports from India to SSA in early 2020, while export volumes to North America increased [29]. North America's stronger purchasing power compared to the African continent made it more attractive to exporters of pharmaceutical supplies. Africa's weak purchasing power is another challenge that impedes its access to pharmaceutical products, given that 70%–95% of the continent's pharmaceutical needs are met from imports [30, 31, 32, 33]. The continent is estimated to have 375 drug manufacturers, serving its 1.1 billion people hence meeting less than 2% of the continent's needs [33, 34, 35]. This contrasts sharply with the 5000 drug manufacturers in China serving its 1.4 billion people and 10,000 drug manufacturers in India serving its 1.4 billion people [34].

The COVID‐19 pandemic also emphasized the limited vaccination manufacturing capacity on the continent. Africa produces only 1% of its vaccines, whereas the 99% is met from import [36, 37]. Vaccines in WHO Africa Regional Office (AFRO) are supplied by the United Nations Children's Fund (UNICEF) through support from The Global Alliance for Vaccinations and Immunization (GAVI), which has shaped markets on the continent to rely heavily on UNICEF [38]. This overdependence coupled with procurement difficulties poses a challenge to establishing sustainable African vaccine production industries [39]. In recognizing the limited local manufacturing capacity, the third pillar of Africa's New Public Health Order addresses this by calling for “expanded manufacturing of vaccines, diagnostics, and therapeutics” [40, 41].

Currently intra‐Africa trade is minimal on the continent, estimated to stand at only 2% during 2015–2019. This is in sharp contrast to 47% in the Americas, 61% in Asia, and 67% in Europe, and similar only to 7% in Oceania which is also heavily reliant on imports [42]. The intra‐Africa trade of medical goods is no different, estimated by the ITC at only 8% [23].

## THE ROLE OF THE AFCFTA ON MEDICAL SUPPLIES ON THE CONTINENT

Prior to 2021, only eight Regional Economic Communities existed to help countries economically integrate and establish regional trade connections [43]. Countries needed an agreement that would allow continent‐wide free trade and ease intra‐Africa trade. The African Continental Free Trade Area (AfCFTA) was introduced for this purpose, making it the largest established free trade area after the World Trade Organization, in terms of the number of countries involved [44]. The agreement was launched in January 2021, serving 1.3 billion people in a US$3.4 trillion economic bloc of 55 countries [44]. The AfCFTA has the potential to change the high medical commodity import dependence of African countries and propagate self‐reliant solutions [45].

Implementing this agreement could help to increase intra‐Africa trade in medical supplies and reform health markets. It will reduce tariffs among member countries and enact policies on regulation and trade facilitation amongst others [33, 44]. Effective implementation of the AfCFTA has the ability to increase the value of intra‐Africa trade and create jobs and revenue [44]. It will also provide the continent with more leverage and purchasing power and boost its ability to enter into more competitive deals within the global market.

**FIGURE 5:**
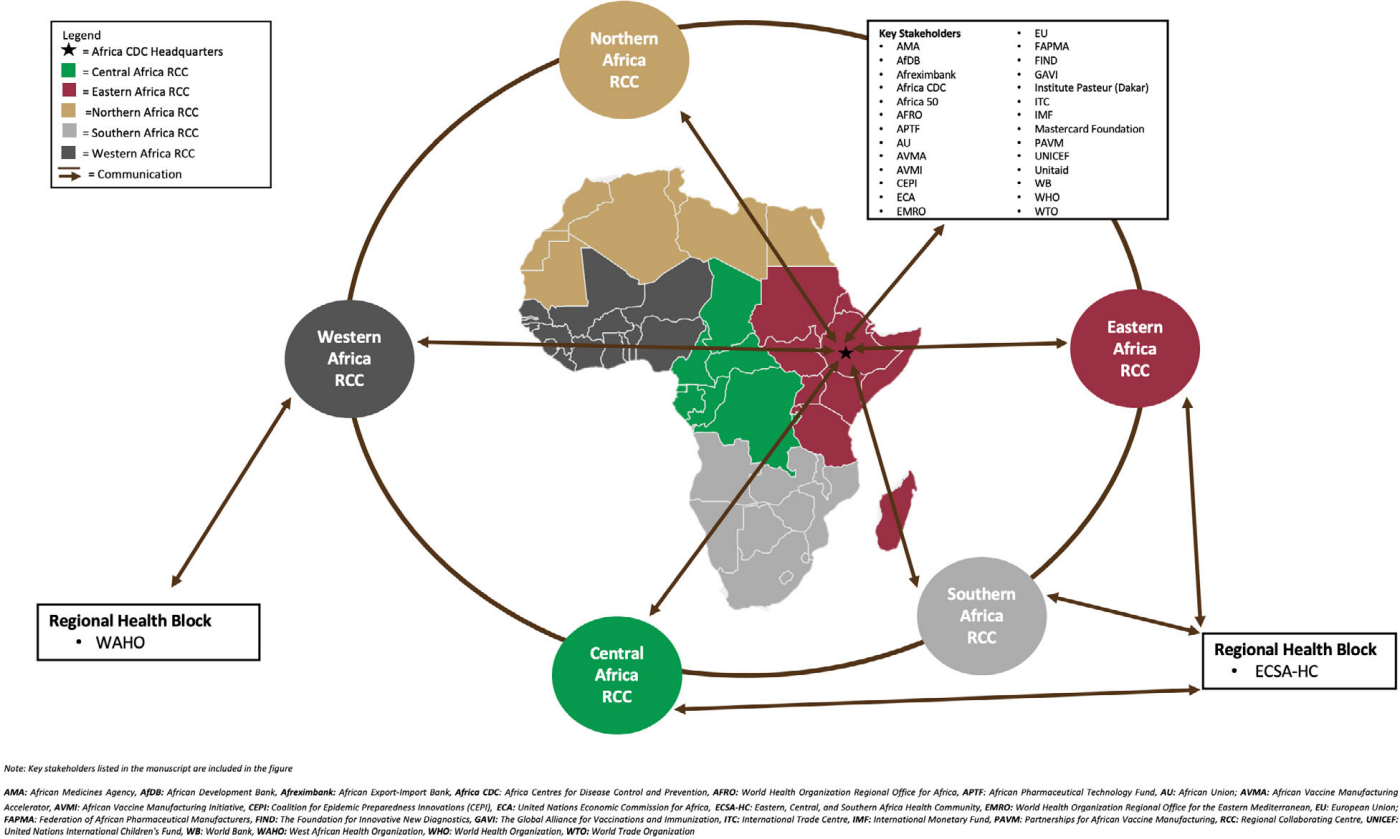
Collaboration among key stakeholders in local manufacturing in Africa with the Africa CDC. *Source*: Adapted from Ref. [47].

Another challenge with the supply of medical supplies is ineffective regional collaboration [46]. The AfCFTA will surmount this challenge by working closely with the Africa CDC's regional collaborating centres (RCC) and with existing regional health communities. Some of these communities are the West Africa Health Organization (WAHO), Eastern Central Southern Africa Health Community in addition to WHO Regional Offices and the United Nations Economic Commission for Africa, which help promote manufacturing and make medical products more accessible [46]. The Africa CDC's RCCs and other regional institutions also need to invest in developing regional hubs to manufacture medical supplies and build value chains that will strengthen intra‐Africa trade. Figure 5 highlights how stakeholders can share information and communicate.

## COVID‐19‐DRIVEN SOLUTIONS TO SUPPLY CHAIN DISRUPTIONS

Due to the limited availability of medical supplies on the continent, it was imperative for local companies to improve their distribution and manufacturing capacities to better meet local demand [48, 49, 50]. For example, Jumia, an e‐commerce giant whose primary market is Africa, used its existing supply chain to help donate face masks to Nigeria, Ivory Coast, Uganda, and Morocco, collaborating with local authorities [51, 52]. In 2022, Jumia also started delivering supplies via drones, improving delivery efficiency [53]. Jumia has the potential to be a model to support the distribution of African‐made products across the continent as AfCFTA is implemented [51].

### Testing

In response to decreased testing kits, Senegal developed a local COVID‐19 test kit at the Pasteur Institute in Dakar in collaboration with Mologic, a UK‐based company [49]. This US$1 test kit was built to withstand challenges of the local environment by requiring minimal lab technology and providing results swiftly, within approximately 10 min [49, 54]. This test uses saliva antigens and/or previous infections by blood antibodies, as opposed to the PCR method which tests sequences of viral RNA [54]. This antigen test method significantly reduces the cost and time to diagnose COVID‐19. Although the antigen test is less accurate compared to more sensitive and specific PCR test, it allows for contact tracing in low‐resource environments [54, 55, 56].

This antigen test in Senegal has the potential to strengthen disease surveillance and control of future outbreaks [57]. In October 2020, only 12 AFRO countries met the key threshold of 10 tests per 10,000 people per week, highlighting the need to scale‐up testing [58]. The Foundation for Innovative New Diagnostics and Unitaid invested in this test to facilitate technology transfer, recognizing the gap in testing capacity in low‐ and middle‐income countries [59]. Since the test's initial manufacture in July 2020, it has been piloted across 14 African countries, and its production capacity is poised to reach 4 million units annually [60].

In Nigeria, the Federal Government worked with LifeBank, a healthcare delivery company, to increase testing capacity [52]. This partnership increased the availability and accessibility of COVID‐19 testing kits for citizens as well as improved data collection. LifeBank has expanded its work to Kenya and Ethiopia, serving over 1000 hospitals [61].

### Masks and personal protective equipment (PPE)

In Kenya, Ultra Red Technologies and other 3D printing companies have been printing 3D face shields and PPE equipment [62]. The face shields are printed by a 2‐part face shield, the first part of which is the 3D‐printed visor and the second part a clear A4 sheet [63]. Ultra Red Technologies is also working to produce 3D‐printed reusable face masks and 3D‐printed ventilator splitters [63]. The ventilator splitters are currently in the testing phase of production in Kenya, undergoing evaluations by medical professionals, including doctors and anesthetists [63].

These local capacity developments have the possibility to be scaled‐up, traded, and implemented by the AfCFTA. This will allow for a more stable supply of medical supplies, which will help to improve health outcomes and strengthen health systems across the continent.

## THE FUTURE OF THE PHARMACEUTICAL MARKET

Recognizing the limited ability to produce pharmaceuticals following the COVID‐19 pandemic, in May 2022, the African Development Bank (AfDB) launched the African Pharmaceutical Technology Foundation (APTF) [64]. The APTF aims to enable regional pharmaceutical production and innovation capabilities [65]. This addresses the objectives of the Federation of African Pharmaceutical Manufacturers Associations, launched in 2013 [66, 67]. Currently, Northern African countries have established pharmaceutical industries which supply the demands of countries in the region, with Egypt producing 90% of its pharmaceutical needs, Morocco caters for 65%, whereas Tunisia and Algeria produce 49% and 30% of their respective needs [68]. Africa's largest pharmaceutical company, Aspen Pharmacare, is based in South Africa with the capacity to produce 6 billion tablets annually. However, it currently supplies drugs to only 22% of SSA [69].

Ethiopia is another country that has invested in local pharmaceutical companies and developed a plan to increase access to locally manufactured quality‐assured medicines, with a goal to meet 85% of its needs [70, 71, 72]. With these investments, the local pharmaceutical market has grown by $337 million from 2015 to 2019, and the market value has been growing by 15% each successive year [72]. The Ethiopia Investment Commission views Ethiopia as a potential hub of pharmaceutical production in the region and even on the continent [72].

In November 2021, the AU's treaty establishing the African Medicines Agency (AMA) came in effect, with the goal to support the Pharmaceutical Manufacturing Plan for Africa [24, 73]. AMA is an AU entity, aimed at standardizing regulatory frameworks, enhancing regulatory supervision, offering technical guidance, and encouraging the adoption of global standards [74]. Currently, 37 out of 55 Members states have signed and/or ratified the AMA treaty [75].

Developing local manufacturing for pharmaceutical products enhances system resilience against global supply chain disruptions, directly addresses the healthcare needs of Africans, and bolsters economic stability. Such local production ensures timely access to vital medications and equipment, key to responding swiftly in health crises. Economic gains from a solid local pharmaceutical industry include job creation, economic growth through industrial multipliers, advancement in research, innovation, and the potential for export, furthering Africa's economic breadth and trade balance. Through collaboration with Africa CDC, there is an opportunity to share knowledge on the production of pharmaceutical products and work with existing pharmaceutical companies to allow for more self‐reliance. With the implementation of AfCFTA and harmonized regulatory frameworks by AMA, pharmaceuticals can be easily transported within the continent.

## THE FUTURE OF THE VACCINE MARKET

In April 2022, Africa was only able to fully vaccinate 17% of its population compared to the world average of 59% [76]. Recognizing inequitable access to COVID‐19 vaccines, the COVID‐19 Vaccines Global Access program was initiated to supply low‐ and middle‐income countries with vaccine doses. This had shortcomings as it could only supply enough doses to vaccinate 20% of the African population [77].

**FIGURE 6:**
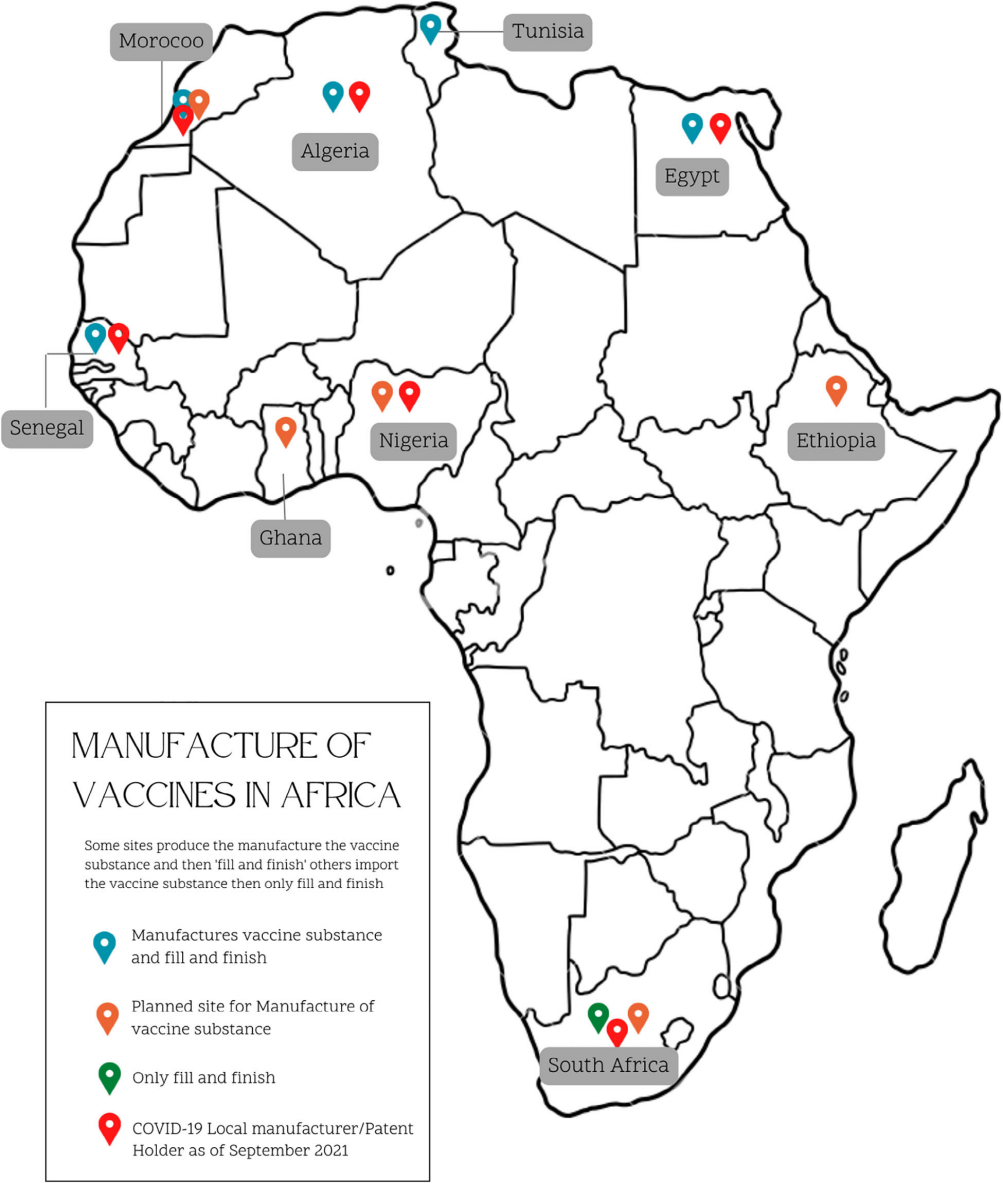
Vaccine manufacturing in Africa in June 2022. *Source*: Adapted from Refs. [80, 81].

Another initiative to remedy vaccine inequities was the COVID‐19 vaccine intellectual patent waiver [78]. This was useful in alleviating the demand‐supply gap, as it led to increased vaccine production on the continent. Unfortunately, the waiver was temporary, and the refusal of high‐income countries to continue to waive intellectual property rights for COVID‐19 vaccines for low‐ and middle‐income countries has been constituted as a human rights violation by the United Nations Committee on the Elimination of Racial Discrimination [79]. Figure 6 highlights African vaccine manufactures and countries now able to produce COVID‐19 vaccines [39].

To further ameliorate the unmet vaccine demand, the AU Member States launched the African Vaccine Acquisition Trust (AVAT), to help the continent achieve its goal of vaccinating 60% of the population [82]. AVAT allowed AU Member States to pool together their purchasing power, reaching a historic agreement in March 2021, purchasing 220 million doses of the single‐shot Johnson & Johnson vaccine [83, 84]. A major benefit of this vaccine is that it is partly manufactured on the continent in Aspen Pharmacare, demonstrating an aspect of self‐reliance [83, 85]. AVAT serves as a model highlighting how, when African countries unite, they can have more leverage and bargaining power in global markets and hence secure necessary supplies needed for health systems.

Recognizing limited vaccination production capacity, the AU and Africa CDC launched the Partnerships for African Vaccine Manufacturing (PAVM) [86]. PAVM aims to manufacture and supply over 60% of the continent's vaccine needs by 2040, potentially growing the market to between US$2.8 and 5.6 billion [87]. Presently, there are seven African manufacturers with vaccine production capabilities in Egypt, Morocco, Senegal, South Africa, Tunisia, and Ethiopia [36, 88]. Private sector contributions are enhancing Africa's vaccine production capabilities, with over 30 vaccine manufacturing projects launched in 14 countries following the COVID‐19 pandemic [87, 89, 90].

The African Vaccine Manufacturing Initiative (AVMI), originally established in 2010, is poised to play a vital role in the development of local vaccine production [91]. Its advocacy efforts over the last decade have led to efforts like the 2015 Vaccine Manufacturing and Procurement in Africa study [92]. AVMI has significantly contributed to understanding and enhancing regional vaccine procurement systems and has established methodologies for vaccine commercialization [93].

With the implementation of the AfCFTA and adequate supply chain networks, vaccine doses can be cheaply and effectively imported across the continent, helping the fight against diseases on the continent [83]. Adopting a regional approach to vaccine production and involving AVMI can shape continental markets and improve access to vaccines in the five RCCs, enabling self‐sustained vaccine production.

## INVESTMENTS FOR LOCAL PRODUCTION

In 2021, Africa CDC launched its inaugural conference to set the agenda for developing a New Public Health Order on the continent [94, 95]. The third Conference of Public Health in Africa had a track and several side events dedicated to the local production of vaccines, diagnostics, and therapeutics [96, 97]. Following the conference, GAVI approved the Africa Vaccine Manufacturing Accelerator, which makes up to US$1 billion available to support sustainable vaccine manufacturing [98]. MasterCard Foundation has also supported Institute Pasteur (Dakar) to expand its workforce for vaccine manufacturing [99].

Without innovation and improvements to manufacturing and the workforce, future pandemic resilience and mitigation will only be as strong as its weakest link—access to global markets and supply chains. There are already ongoing efforts to strengthen health systems infrastructure and the workforce through initiatives launched by Pan‐African institutions such as Afreximbank, AfDB, Africa50, and Africa CDC [94, 95, 100]. African governments also need to prioritize increasing investments in the health sector. Continuing these investments and initiatives and engaging with health ministers, relevant stakeholders, and the affected populations will lead to more resilient health systems on the continent.

## CONCLUSION

The COVID‐19 pandemic has highlighted Africa's overdependence on the imports of medical goods and access to global markets and supply chains. This has led to investments in production capabilities on the continent. It has also revealed the capacity of African countries to innovate and develop homegrown solutions to medical emergencies when the right policy environment and support exist. The implementation of AfCFTA provides an opportunity for increased local production of medical supplies, which can be traded within the African continent and the expansion of COVID‐19‐driven solutions to alleviate supply chain disruptions. AfCFTA has the potential to enable African countries to be better prepared for future pandemics and meet the health needs of their citizens. This agreement also equips the continent with more leverage, bargaining power, and purchasing power in global markets. Increasing the intra‐Africa trade of medical supplies could help strengthen the medical supply chains, thereby strengthening health systems and encouraging a Pan‐African response to health emergencies.

## AUTHOR CONTRIBUTIONS

Jonta Kamara contributed to the conception, investigation, drafting, editing, review, and visualization of the work. Ukeme Essien contributed to the drafting, editing, review, and visualization of the work. Alain Labrique contributed to the editing and review of the work.

## CONFLICT OF INTEREST STATEMENT

None of the authors have conflicts of interest to disclose.

## DISCLAIMER

The views expressed are the authors’ and do not represent the views of organizations they are affiliated with. Organizations have different definitions of which countries are considered a part of sub‐Saharan Africa.

## FUNDING INFORMATION

No funding was received for this research.

## Data Availability

Data sharing is not applicable to this article as no new data were created or analyzed in this study.
